# Medical and Physical Intelligence

**Published:** 1821-02

**Authors:** 


### Medical and Physical Intelligence.

TRANSACTIONS OF SCIENTIFIC SOCIETIES.

*ROYAL SOCIETY of London.—Nov.* 23. A LETTER to the President from the Earl of Morton was read, communicating a curious fact in natural history.

The Earl of Morton had received, some years since, a male *quagga* from the Cape of Good Hope. From this and an Arabian blood chestnut mare a female hybrid was produced, which possessed indications, both as to colour and form, of her mixed breed. In the possession of another person, the same mare was afterwards bred from by an Arabian horse: the result was singular; as, although it had the general appearance of the Arabian breed, it bore, in several respects, characters which belong to the quagga, such as stripes and marks on the body, and in the hair of the mane.

---

*Case of Extirpation of the Thyroid Gland,* by Dr. KLEIN, of Stuttgardt.—A BOY, eleven years of age, deaf and dumb, and of a very delicate constitution, had, from his infancy, a circumscribed tumor situate on the left side of the neck, about the size of a nut, and which had gradually increased in bulk. After having been put under the care of several practitioners, he was brought to Dr. Klein. The tumor commenced on the left side, at the margin of the jaw, occupied the whole of the left side of the neck, as far as from the larynx to behind the ear, and down as low as the third rib. Vessels of the size of the finger run over its surface: it was movable with difficulty, rather because of its weight than of its adherence to the parts beneath. It was a little irregular, or tuberculated, in its construction; and the pulsation of the arteries which penetrated its interior could be distinctly felt in several parts. Its removal by the knife having been determined on, the patient was laid on a table, so that his head, shoulders, and legs, might be most effectually secured by assistants. On making the necessary preliminary incisions in the integuments, the veins, which were much dilated, were divided, and threw out about half-a-pound of blood; but they were soon secured by compression with the fingers, as well as the subclavian artery. After the flaps of the integuments were dissected aside, the tumor was forcibly drawn outwards; and, using sometimes the fingers and sometimes the scalpel, or its handle, it was separated from the larynx and trachea. The operation occupied a minute and a half; but Dr. Klein was surprised to find no hemorrhage, even from the thyroid arteries, which were, of course, divided. On examining the patient, however, he was found to be insensible; and, though every proper means was used for three-quarters of an hour, it was found impossible to restore him to life. On opening the body, it was found that neither the carotid artery, the par vagum, the jugular vein, nor the trachea, had been injured; but all the vessels of the dura mater and the brain were gorged with blood:

there was a large quantity of serum in the ventricles. "I think myself authorized," says Dr. Klein, "to conclude that the patient died of apoplexy occasioned by the revolution in the circulation which the operation had caused. The tumor itself was found by the thyroid gland, which had suffered disorganization throughout its whole structure, excepting a small portion on the right side. It weighed two pounds, and its base was of about six inches diameter in every direction. It was formed of a solid, lardaceous, reddish substance, divided into several lobules."

In regard to the cause of death in this case, we would suggest to others the consideration whether or not *moral* circumstances had some influence in causing it. The agitation and suffering of the boy,—being deaf and dumb, and consequently incapable of understanding the object or necessity of the operation,—must have been extremely intense. We doubt whether such an operation on such a subject was justifiable.

---

Professor Dupuy, of the Veterinary School of Alfort, has made some further experiments (noticed in the *Compte rendu des Travaux de l'Ecole royale d'Alfort, dans la séance du* 4 *Nov.* 1820,) on the inoculation of the *scab* in sheep, which tend to establish very satisfactorily the utility of the practice. At a time when this disease was prevalent in an extensive flock, and of a very confluent form, he inoculated two hundred, of which only three died; whilst the mortality in those who took the disease naturally was in the proportion of one in five. There were, besides, reasons for a supposition that the three inoculated sheep which died had taken the disease naturally previously to, or about the time of, the operation.

We are not aware whether or not inoculation for the *scab* has been practised in England, and we have not the means at this moment, when these observations are required for the press, of ascertaining this point; though we have some reason to think that, if it had been practised, it would not have escaped our knowledge; but it has often been resorted to in France, in Italy, and in Hungary, and always with the most favourable results,—the inoculated animals escaping the disease by infection, and having it, almost universally, in so mild a form as to be free from danger of fatal consequences. We may remark here, that the supposition, which had at one time gained some degree of credit, of the identity of cow-pox and the scab, has been proved to be incorrect; and it has also been found that cow-pox will not preserve sheep from the scab.

The most favourable parts for the inoculation, for many reasons, are those points of the chest which are devoid of wool. Three or four punctures should be made, to secure the success of the operation: more would produce an inconvenient local affection.

We some time since noticed in this Journal, (April 1819,) that Mr. Wilkinson, of Newcastle, had found signs of inflammation of the spinal marrow, in several instances, in horses which had died of tetanus: Professor Dupuy says, that he has always found such an affection of this part after death from this disease. He adds to this interesting fact the following observations:—A troop of cows, consisting of fifty in number, was attacked with an epizooty which was prevalent at the time, (in 1814;) when, on the 3d of June, there occurred a violent storm of thunder and lightning. On the following day, fifteen of the animals, which had been previously diseased, died on the next day, almost suddenly. Prof. Dupuy dissected them. He only found considerable disease of the spinal marrow, which was much softened, with effusion of serum into the theca.

---

*Sulphate of Platinum a Test for Gelatine.*—Mr. E. Davy recommends the use of the sulphate of platinum in detecting small quantities of gelatine. From comparative experiments made with it, and astringent infusions, he found, that when the quantity of gelatine was so small as not to be effected by strong infusions of oak-bark, nut-galls, or catechu, still there was an immediate precipitate on adding the sulphate of platinum. Where the proportion of gelatine was so reduced as not even to affect sulphate of platinum at first, the precipitate was immediately produced on boiling the fluid.

The different astringent infusions, as of oak-bark, nut-galls, catechu, &c. do not act uniformly on the various kinds of gelatine: thus, an infusion of catechu would produce no precipitate in solutions of paper-hangers' size; but the sulphate of platinum acts equally on all kinds of size, and throws down precipitates which appear to be always similar, not being affected even by the presence of free acid in the solution.—*Journal of Science, &c.*

---

*On Iodine, and its Existence in Sponge.*—We inserted, in a late Number, a paper by Dr. Coindet, on the use of iodine as a remedy for bronchocele; and gave, at the same time, an account of Mr. Fife's discovery of its existence in sponge: we now find that Mr. Straub, of Hofwyl, as early as December 1819, appears to have shown its existence in this substance, and proposed the use of it instead of the *spongia usta* in medicine. In order to obtain the iodine from sponge, the latter, after being burnt, was washed with water, and the solution decomposed by sulphuric acid; and in this way so much was obtained from half an ounce of sponge as to confirm the ideas previously entertained, that its medicinal properties were owing to this substance.

Mr. Straub recommends trials of preparations of iodine in medicine, and thinks that, where salts formed from it cannot be obtained, an alcoholic extract of burnt sponge is much to be preferred to the burnt sponge itself.

Mr. Straub also asserts the existence of iodine in turf. He was led to examine this substance in consequence of the peculiar odour he observed in the neighbourhood of those buildings where turf is burnt. Repeated experiments confirmed this conjecture; and, by acting on two pounds of turf, abundant evidence of the existence of iodine in it may be obtained. It was found also in the cinders of the *helmintocorton*, though in very small quantities.

### METEOROLOGICAL JOURNAL.

By Messrs. WILLIAM HARRIS and Co. 50, Holborn, London.

*From December 20 to January 19, inclusive.*

| Day of Month. | Moon. | Rain gauge | THERM. | | | BAROM. | | DeLuc's Hygro. | | WIND. | | ATMOSPHERIC VARIATION. | | |
|---|---|---|---|---|---|---|---|---|---|---|---|---|---|---|
| Dec | | | | | | | | | | | | | | |
| 20 | | ·03 | 44 | 50 | 49 | 30·38 | 30·20 | 66 | 67 | WSW | SW | Cloudy | Rain | Cloud. |
| 21 | | | 49 | 50 | 43 | 30·11 | 30·17 | 65 | 63 | W | WNW | Cloudy | | Fine |
| 22 | | | 38 | 47 | 42 | 30·15 | 30·09 | 61 | 62 | WNW | WNW | Cloudy | | Rain |
| 23 | | ·04 | 40 | 45 | 33 | 30·00 | 29·90 | 62 | 61 | NE | ENE | Cloudy | Sho'ry | Cloud. |
| 24 | | ·02 | 33 | 36 | 33 | 29·88 | 29·87 | 59 | 57 | ENE | ENE | Cloudy | | |
| 25 | | | 33 | 36 | 30 | 29·84 | 29·84 | 56 | 56 | ENE | ENE | Cloudy | | |
| 26 | | | 31 | 33 | 31 | 29·78 | 29.90 | 57 | 58 | E | E | Cloudy | | |
| 27 | ☽ | | 32 | 33 | 31 | 29·92 | 29·93 | 58 | 60 | NE | ENE | Cloudy | | |
| 28 | | | 31 | 31 | 27 | 29·94 | 30·00 | 59 | 59 | NNE | NE | Fine | | |
| 29 | | | 28 | 29 | 26 | 30·00 | 29·93 | 56 | 57 | NE | NE | Fine | | |
| 30 | | | 28 | 29 | 25 | 29·97 | 29·97 | 57 | 56 | E | NE | Cloudy | Fine | |
| 31 | | | 27 | 30 | 28 | 29·95 | 29·93 | 55 | 57 | NE | ENE | Fine | | |
| Jan. | | | | | | | | | | | | | | |
| 1 | | | 31 | 32 | 27 | 29·88 | 29·86 | 57 | 56 | ENE | ENE | Cloudy | | |
| 2 | | | 28 | 30 | 27 | 29.74 | 29·60 | 58 | 57 | SE | E | Cloudy | | |
| 3 | | | 28 | 31 | 27 | 29·46 | 29·35 | 57 | 57 | E | NE | Cloudy | | |
| 4 | ● | | 27 | 31 | 30 | 29·41 | 29·40 | 58 | 58 | NE | ENE | Cloudy | | |
| 5 | | ·05 | 31 | 32 | 33 | 29·35 | 29·23 | 59 | 62 | NE | E | Cloudy | Light Sn | Snow |
| 6 | | ·26 | 36 | 40 | 35 | 29·20 | 29·27 | 63 | 65 | E | NNE | Rain | | |
| 7 | | ·11 | 37 | 39 | 38 | 29·27 | 29·23 | 67 | 67 | E | E | Rain | Cloud. | |
| 8 | | ·15 | 40 | 43 | 35 | 29·21 | 29·19 | 66 | 67 | E | E | Fine | Cloud. | Rain |
| 9 | | ·03 | 39 | 45 | 40 | 29·12 | 29·10 | 63 | 65 | SSW | SW | Fog | | |
| 10 | | ·13 | 41 | 46 | 40 | 29·16 | 29·11 | 65 | 67 | SW | E | Cloudy | | Rain. |
| 11 | ☾ | ·28 | 45 | 49 | 40 | 29·16 | 29·20 | 67 | 65 | E | ENE | Rain | | |
| 12 | | ·19 | 43 | 53 | 39 | 29·30 | 29·40 | 65 | 65 | SW | SE | Rain | | Cloud. |
| 13 | | ·09 | 45 | 50 | 39 | 29·57 | 29·23 | 60 | 63 | WSW | SE | Fine | Cloud. | Rain |
| 14 | | ·31 | 43 | 45 | 38 | 29·15 | 30·08 | 65 | 67 | E | ENE | Rain | | Fine |
| 15 | | ·13 | 41 | 45 | 39 | 30·00 | 30·07 | 67 | 65 | E | E | Rain | | Cloud. |
| 16 | | ·09 | 43 | 47 | 41 | 30·11 | 30·17 | 60 | 60 | SE | ESE | Fine | Cloud. | Rain |
| 17 | | ·11 | 47 | 50 | 45 | 30·20 | 30·21 | 64 | 62 | E | SW | Rain | Cloud. | |
| 18 | ○ | | 51 | 54 | 48 | 30·18 | 30·23 | 65 | 63 | SW | WSW | Cloudy | | |
| 19 | | ·08 | 50 | 52 | | 30·31 | 30·27 | 63 | 61 | SW | SW | Rain | Cloud | |

The quantity of rain fallen in the month of December, is 1 inch and 61-100ths.

